# Morphological diversity in the foraminiferal genus *Marginopora*

**DOI:** 10.1371/journal.pone.0208158

**Published:** 2018-12-26

**Authors:** Willem Renema

**Affiliations:** Naturalis Biodiversity Center, RA Leiden, the Netherlands; Universita degli Studi di Urbino Carlo Bo, ITALY

## Abstract

Benthic foraminifera, and certainly symbiont-bearing (large) benthic foraminifera are generally considered to have large geographic ranges in combination with significant ecomorphological variation. With the advance of molecular phylogenetic approaches, supported or preceded by detailed morphological studies, it was demonstrated that this view needs to be reevaluated. In this paper I evaluate the morphology of five *Marginopora* populations from around the Coral Sea by microCT-scanning. I argue that ecomorphological and ontogenetic variation is smaller than geographic variation in morphology. This forms the basis for the description of three new species, *M*. *santoensis* nov. spec., *M*. *charlottensis* nov. spec., *M*. *orpheusensis* nov. spec. Quantitative morphological variation between *M*. *rossi*, *M*. *orpheusensis* nov. spec. and *M*. *charlottensis* nov. spec. is overlapping, but each species has unique morphological characters supporting recognition as new species. Support to distinguish the deep living (*M*. *rossi*, *M*. *charlottensis* nov. spec., *M*. *orpheusensis* nov. spec.) and shallow living (*M*. *vertebralis*) *Marginopora* populations as separate species is strong, but not enough molecular phylogenetic data are available to test the three new deep-living species on the Great Barrier Reef hypothesis. However, detailed understanding of ecophenotypic variation in *M*. *santoensis* nov. spec. supports the conclusion that it is unlikely that ecophenotypic variation can explain the morphological variation between the three species. I argue that the number of species in this genus is underestimated, and that there are at least five species in the Coral Sea area alone.

## Introduction

Foraminiferal taxonomy, as in other groups of organisms, have been subject to waves of taxonomic splitters and lumpers. Initially many populations were described as new species based on sometimes marginal morphological differences. In later days, however, the importance of ecophenotypic variation was emphasized, and what was previously interpreted as multiple species were lumped into single species with wide geographical ranges and considerable morphological variation [1]. This tendency was especially strong for taxa that are widely used as indicators for either stratigraphy or environmental reconstruction.

With the advent of molecular techniques, independent tests of the value of morphological characters became available. These methods revealed that some taxa are truly wide ranging, covering one or more entire ocean basins, but that species with small geographic distribution occur as well [2]. These and related results demonstrated that several characters previously used for generic or suprageneric classification were the result of convergent evolution [3,4]. Examples include the evolution of secondary chamberlets in Nummulitidae [4], and the evolution of a complex internal structure in the Soritinae [3]. Within the Soritinae it was long assumed that the evolution of the median layer was reflected in the apertural face, and increased linearly from *Sorites* with a single row of apertures, into *Amphisorus* with a double row of apertures, and finally into *Marginopora* with multiple rows of apertures (e.g., [5,6]). However, the structurally complex genera *Amphisorus* and *Marginopora* evolved independently from the structurally simple genus *Sorites* [3].

At the same time, morphological approaches also demonstrated the presence of species with very restricted ranges, for example in the genus *Amphisorus* [6, 7]. Until the description of *Amphisorus kudikajimaensis* (originally placed in the genus *Marginopora*) from southern Japan [6], this was considered to be a monotypic genus with a circumtropical distribution. Later a third species of *Amphisorus* was found in the Great Barrier Reef [7]. These two finds were based on morphology, but have later been confirmed using molecular techniques [3,7,8]. Based on these new insights, morphological characters have been discovered that define these genera. *Amphisorus* has two rows of elongated marginal apertures and in some taxa one or more layers of irregularly shaped median apertures [7]. In *Marginopora* on the other hand both the marginal and median apertures are more or less round and rimmed.

Over the years I have collected *Marginopora* from a large number of places. What was striking is that *Marginopora vertebralis* is a large, often wrinkled species occurring in the uppermost reef slope and reef crest on solid or sometimes phytal substrates in highly exposed reefs (e.g. [9,10,11]). However, in several localities around the Coral Sea, specimens of *Marginopora* also live in very calm, reef base and inter reef environments, often on a sandy substrate [8]. These populations can attain a larger diameter than typical *Marginopora vertebralis*, and some populations frequently lose their embryonic apparatus and the initial whorls to leave a hole in the center of the test.

**Fig 1:**
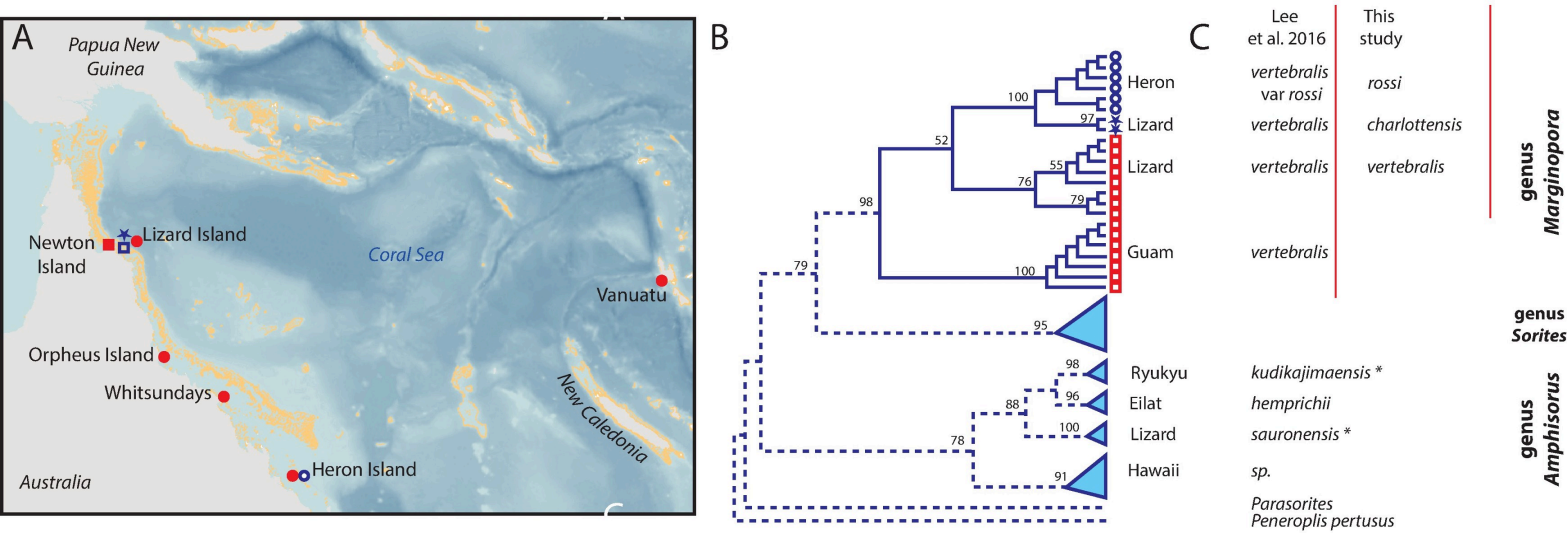
**Summary diagram showing the research area (A), the phylogram of [8]; B), and the re-interpretation of the results of [8] in this study (C**). In A and B circles indicate collections made at the reef base or inter reef, squares collections made at the reef flat, and open symbols include collections included in [8], closed symbols collections included in this study. Finally, the stars indicate the two specimens from Heron island sequenced by [12].

One of these populations was included in a combined molecular and morphological study [8], concluding that morphological and structural differences exist between the deep-living population around Heron Island, and shallow-living populations on Lizard Island (both Great Barrier Reef; Fig 1). However, based on their molecular results, they place both populations in a single species (Fig 1B). There are three striking consequences of this conclusion:

1. Within the analyzed specimens of *Marginopora*, there is a strong structuring based on geography (Fig 1). Only two specimens analyzed by [12] from Lizard Island group next to the Heron Island specimens of [8].
2. Within the *Marginopora* specimens analyzed by [8] there is a strong structuring based on habitat (Fig 1). Once again, the two specimens from Lizard Island analyzed by [12] group next to the specimens from the Heron Island reef base.
3. The observed genetic variation in monotypic *Marginopora* is much larger than in *Amphisorus*, in which at least three accepted species show fewer genetic difference (Fig 1). Interestingly, two out of four of the *Amphisorus* populations (*A*. *kudikajimaensis* and *A*. *sauronensis*) have a median skeleton, whereas *A*. *hemprichii* and *A*. sp. (Hawaii) lack a median skeleton.

Based on these observations, I will first assess morphological differences of four populations of deep living and a four populations of shallow living *Marginopora* from the Coral Sea, using microCT scans of the shells. This is followed by a discussion on how my morphological observations fit into the available molecular phylogenetic results of [8, 12, 13].

## Methods

### Material

Samples were collected by SCUBA-diving (*M*. *orpheusensis* nov. spec., *M*. *rossi*, *M*. *santoensis* nov. spec.), snorkeling (*M*. *vertebralis*) or by grab sampling (*M*. *charlottensis* nov. spec.) during a sedimentological survey with the R.V. Kirby. Specimens from the reef crest of Orpheus Island and Lizard Island were provided by Martina de Freitas Prazeres and Sven Uthicke. All specimens used in this study were alive at the time of collecting. Live specimens can be recognized based on the brownish, greenish, or reddish grey color of the symbionts. All specimens are A-forms, unless otherwise mentioned. For the samples collected within the Great Barrier Reef Marine Park, no permits were required for collecting foraminifera at the locations included in this study, because this is regarded as limited impact activities for which no permits are needed (http://www.gbrmpa.gov.au/zoning-permits-and-plans/permits) by the Great Barrier Reef National Park Authority.

### Micro computed tomography scanning and data processing

Shells were scanned using a Brüker/Skyscan 1172 at 1–3 μm voxel size. Settings used for scanning were binning: 4000 x 2672 pixels; no filter; 60 kv, camera rotation steps of 0.1–0.25 degrees, and an exposure time of 700–1100 ms, depending on the size of the specimen and the resolution of the scan. Original X-ray projections were reconstructed using NRecon. Visualisations of the interseptal space were made using Avizo 9.0.

### Nomenclatural acts

The electronic edition of this article conforms to the requirements of the amended International Code of Zoological Nomenclature, and hence the new names contained herein are available under that Code from the electronic edition of this article. This published work and the nomenclatural acts it contains have been registered in ZooBank, the online registration system for the ICZN. The ZooBank LSIDs (Life Science Identifiers) can be resolved and the associated information viewed through any standard web browser by appending the LSID (Life Science Identifiers) to the prefix “http://zoobank.org/”. The LSID for this publication is: urn:lsid:zoobank.org:pub:0105F275-B9D8-40A5-A807-0B73F3CAB114. The electronic edition of this work was published in a journal with an ISSN, and has been archived and is available from the following digital repositories: PubMed Central, LOCKSS.

## Results

### Systematics

Class Foraminifera d’Orbigny, 1826 [14]

Order Tubothalamea, Pawlowski, Holzman, & Tyszka, 2013 [15]

Family Soritidae Ehrenberg, 1839 [16]

Subfamily Soritinae Ehrenberg, 1839 [16]

Genus *Marginopora* Quoy & Gaimard in Blainville, 1830 [17]

Type species: *Marginopora vertebralis* Quoy and Gaimard, 1830 [17]

### Diagnosis

**Fig 2:**
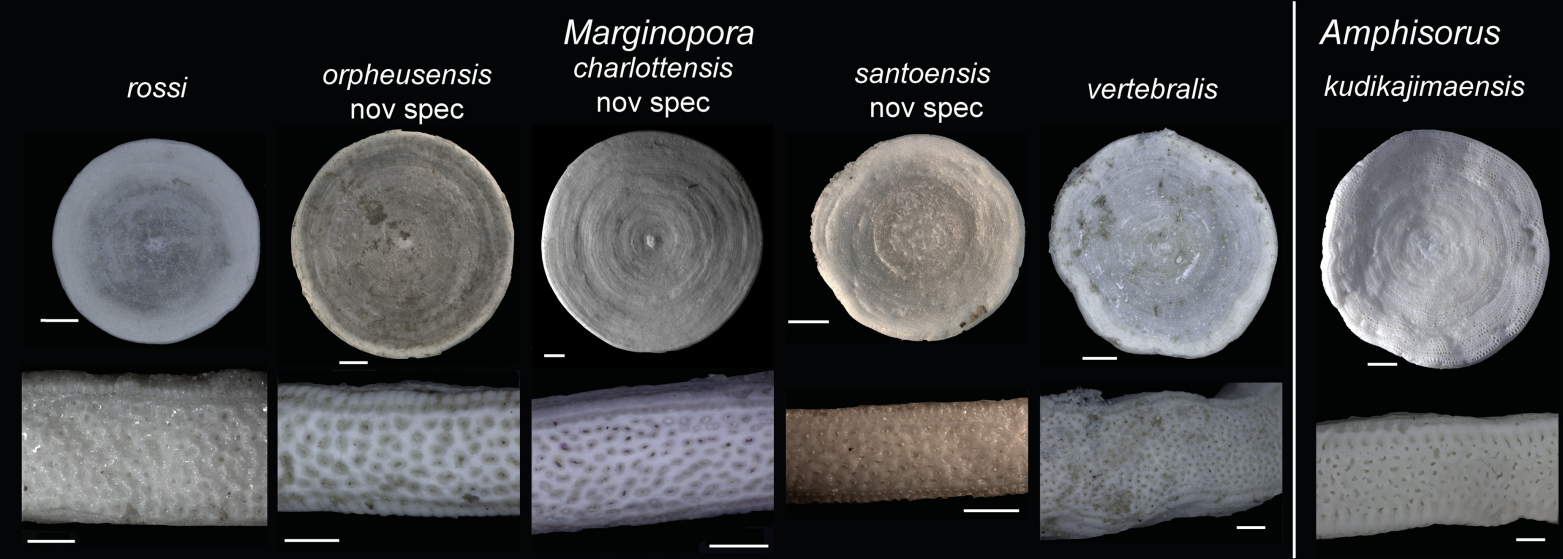
External views of the five species of *Marginopora* discussed in this paper, and *Amphisorus kudikajimaensis* as a comparison. Note the larger apertures, of which the median apertures are irregularly shaped in *A*. *kudikajimaenis*, whereas in *Marginopora* the median apertures are small and round to elongated. The marginal chamberlets are narrow in *Marginopora* compared to *A*. *kudikajimaensis*. Scale bars in upper row represent 1mm, in the lower row 200μm.

Large, round and flat soritids, with two rows of marginal chamberlets, two annular passages and median chamberlets, both the median and marginal apertures are small round to slightly elongated and rimmed (Fig 2). The rimmed nature of the apertures is most clearly developed in the marginal apertures, the median apertures are positioned in depressions. In vertical section the marginal chamberlets are narrow, less than 15% of the shell thickness in the outer chambers. *Amphisorus* is the only other soritid that has both median and marginal apertures in at least some of the taxa (for example A. *kudikajimaensis*, *A*. *sauronensis*), but the median apertures are not rimmed and more irregularly shaped, and the marginal apertures can be elongated to slit like (Fig 2).

**Fig 3:**
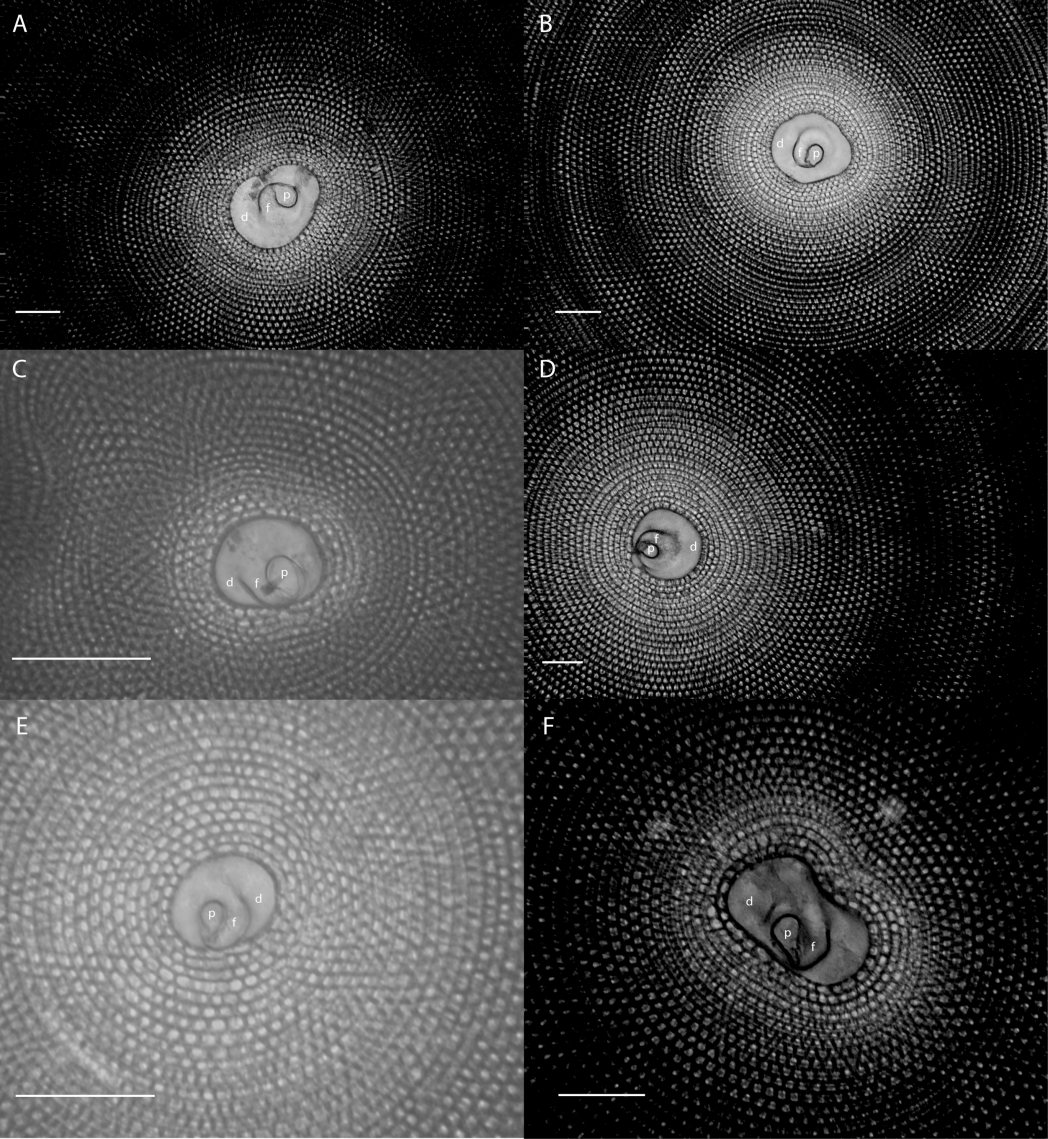
X-ray projections of the center of the shell of *Marginopora* spp. A) *M*. *rossi* (RGM.1352064) with the deuteroconch (d) completely enveloping the protoconch (p) and flexostyle (f), B) *M*. *charlottensis* nov. spec. (RGM.1352050); C) *M*. *vertebralis* (RGM.1352105); D) *M*. *orpheusensis* nov. spec. (RGM.1352059); E) *M*. *santoensis* nov. spec. (RGM.1352122); F) *M*. *charlottensis* nov. spec. (RGM.1352055). Especially in the populations with larger deuteroconchs, irregularly shaped deuteroconchs are frequent, resulting in more variance in the deuteroconch diameters. Scale bar 500μm.

**Fig 4:**
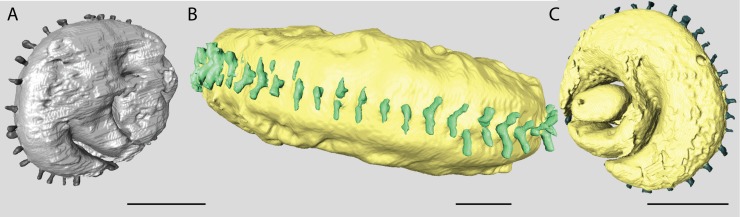
**Reconstructions of the protoconch and deuteroconch of A) *Marginopora vertebralis;* B) *M*. *rossi* and C) *M*. *santoensis* nov. spec**. Note the single row of I-shaped stolons in *M*. *vertebralis* and *M*. *santoensis*, and the Y-shaped stolons in *M*. *rossi*. Scale bars are approximately 200 μm.

**Fig 5:**
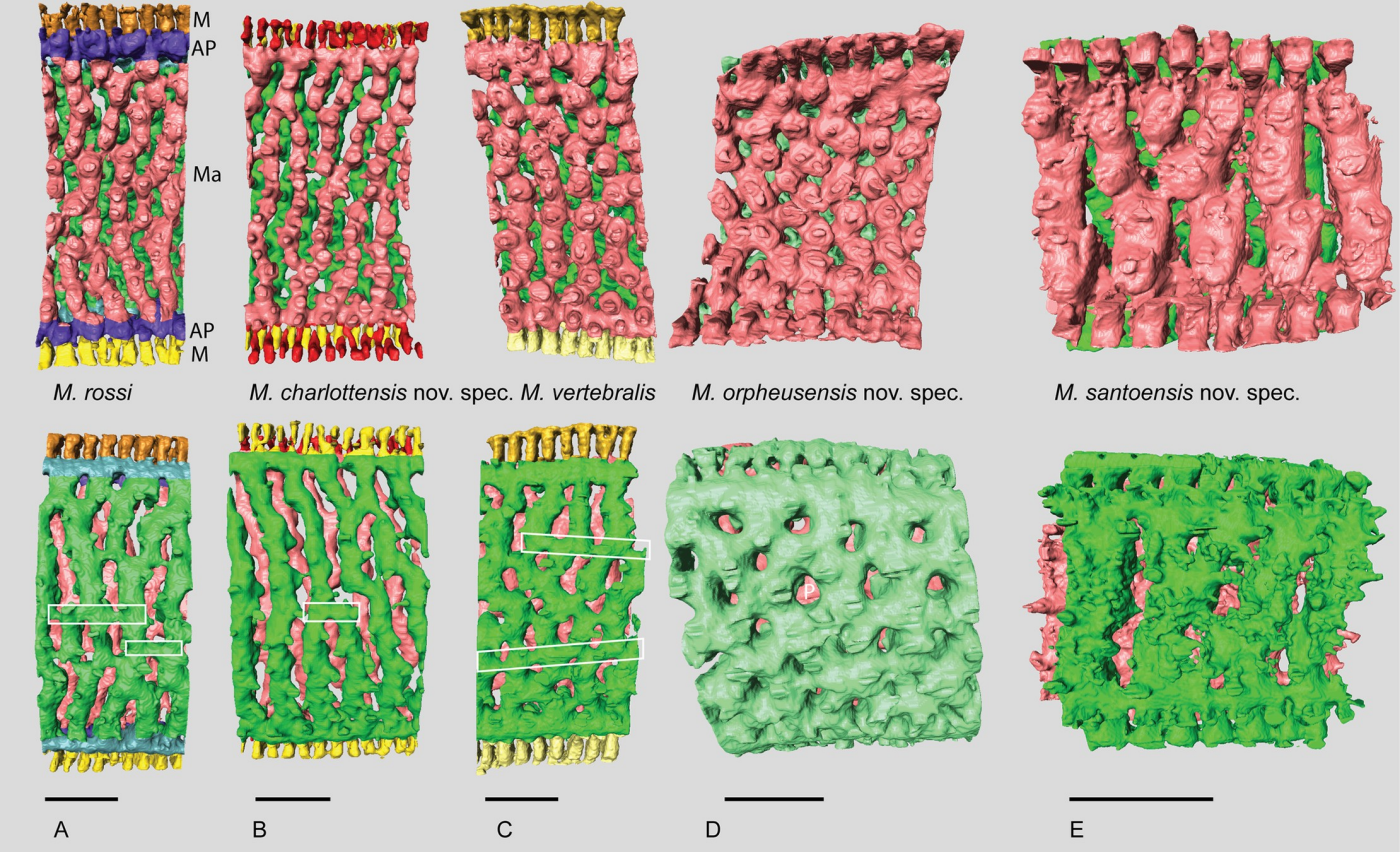
Micro-computed tomography of *Marginopora* with the shell removed, showing the interseptal space of two subsequent chambers at a thickness of ~10 stolon planes, demonstrating the structural differences between the five species. The top row is projected looking from the inside out, the bottom row shows the same chambers looking from the outside in. Pink chamber is the innermost, green chamber the outermost of the reconstructed chambers. In A-C the marginal chambers have been highlighted in yellow. A) *M*. *rossi*, showing frequent annular connections (white squares) of 2–3 adjacent chamberlets. Note the three way differentiation within the chamber: marginal chamberlets (M, yellow) connection to the annular passage of the current and preceding whorl on either side of the shell, the annular passage (AP, blue and purple) which is partitioned by septula, and the marginoporid structure (Ma) containing the median chamberlets and the radially oblique stolon planes (RGM.1352065). B) *M*. *charlottensis* nov. spec. showing regular chamberlet width and the very rare annular connections (white squares), resulting in a very regular looking shell (G466207). C) *M*. *vertebralis* showing the irregular shape of the chamberlets, the frequent annular connections sometimes connecting up to 5 adjacent chamberlets (white squares) (RGM.1352105). D) *M*. *orpheusensis* nov. spec. showing the high interconnectedness of the chamberlets. Apart from the radially oblique stolon planes, chamberlets are also connected by stolon planes within the chamberlets at ~45° to the annular passage (G466208). E) *M*. *santoensis* nov. spec. showing the regular chamberlet shape and the low level of interconnectedness (RGM.1352067). Scale bars are approximately 200 μm.

The embryonic apparatus of *Marginopora* consists of a protoconch and a flexostyle partially or entirely encircled by the deuteroconch (Fig 3). The deuteroconch is connected to the first chamber by multiple radial stolons (Fig 4). The deuteroconch is surrounded by annular chambers divided into chamberlets. Fully developed, each chamber consists of multiple rows of median chamberlets, on either side connected via an annular passages partitioned by septula, to median chamberlets (Fig 5). The median chamberlets are arranged along oblique apertural axes with respect to the radial direction, which cross over in neighboring stolon planes, and alternating in direction from one stolon plane to the next (marginoporid structure as described in [18]). Apart from the radial oblique stolon axes, adjacent chamberlets in the marginoporid structure can be connected by annular connections within the chamber (Fig 5). The chamberlets are separated by interseptal ridges between the septa that can be straight, bifurcating, or disjunct leading to a reduction to pillars in some species (Fig 5). In the earliest formed chambers this tripartite structure is usually not yet developed, in which case each chamber consists of an annular passage with chamberlets on either side. These are morphologically different from marginal chamberlets since they only connect to the annular passage of a single chamber, whereas marginal chamberlets connect both to the annular passage last formed chamber and the that of the preceding chamber.

*Marginopora vertebralis* Quoy & Gaimard in Blainville, 1830 [17].

### Material

RGM.1352103-111. Numerous specimens collected on the reef flat and reef crest of Newton Island (Princess Charlotte Bay). 25 specimens were microCT-scanned. Fifteen specimens from Orpheus Island (Pioneer Bay, 3 m depth on rubble) collected in November 2017 by Martina de Freitas Prazeres, and an additional seven specimens from the same location but collected in 2011 by Sven Uthicke.

This species has a wide distribution across the West Pacific. However, based on the sequencing results presented in [8, 12, 13] it is likely that there are several cryptic species within this taxon (Fig 1).

### Description

#### External morphology

**Fig 6:**
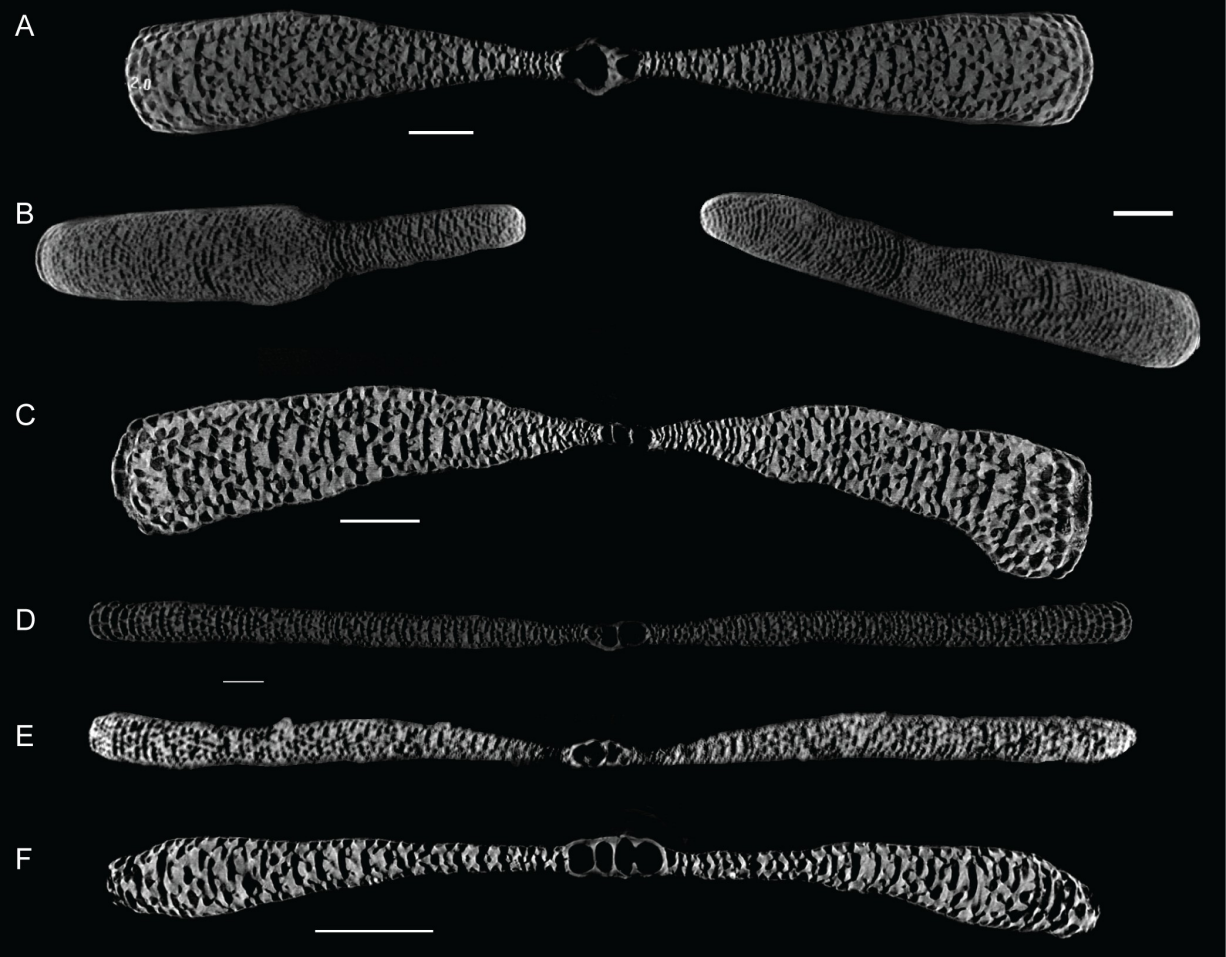
Virtual vertical thin sections through the A-forms of *Marginopora* studied here. A) *M*. *rossi*, *s*mall specimen, showing the embryonic apparatus and the gradual increase in thickness (RGM.1352063; scale bar 500 μm). B) *M*. *rossi*, *l*arge specimen, which lost the embryonic apparatus and initial whorls (RGM.1352066; scale_bar 1 mm). C) *M*. *vertebralis* (RGM.1352103; scale bar 500 μm). D) *M*.*orpheusensis* nov. spec. (RGM.1352059; scale bar 500 μm). E) *M*. *charlottensis* nov. spec. (RGM.1352045); Scale bar 500 μm). F) *M*. *santoensis* nov. spec. (RGM.1352101; Scale bar 500 μm).

Diameter of the microsphere up to 17 mm, rapidly increasing in thickness in the initial chambers. Up to 1.5 mm thick, irregular and often with a wrinkled periphery (plicated in [8]). The macrosphere can grow up to a diameter of 6 mm, and rapidly increases in thickness during growth (Fig 6). The thickest specimens achieve a thickness of 0.8–0.9 mm.

#### Embryonic apparatus

**Fig 7:**
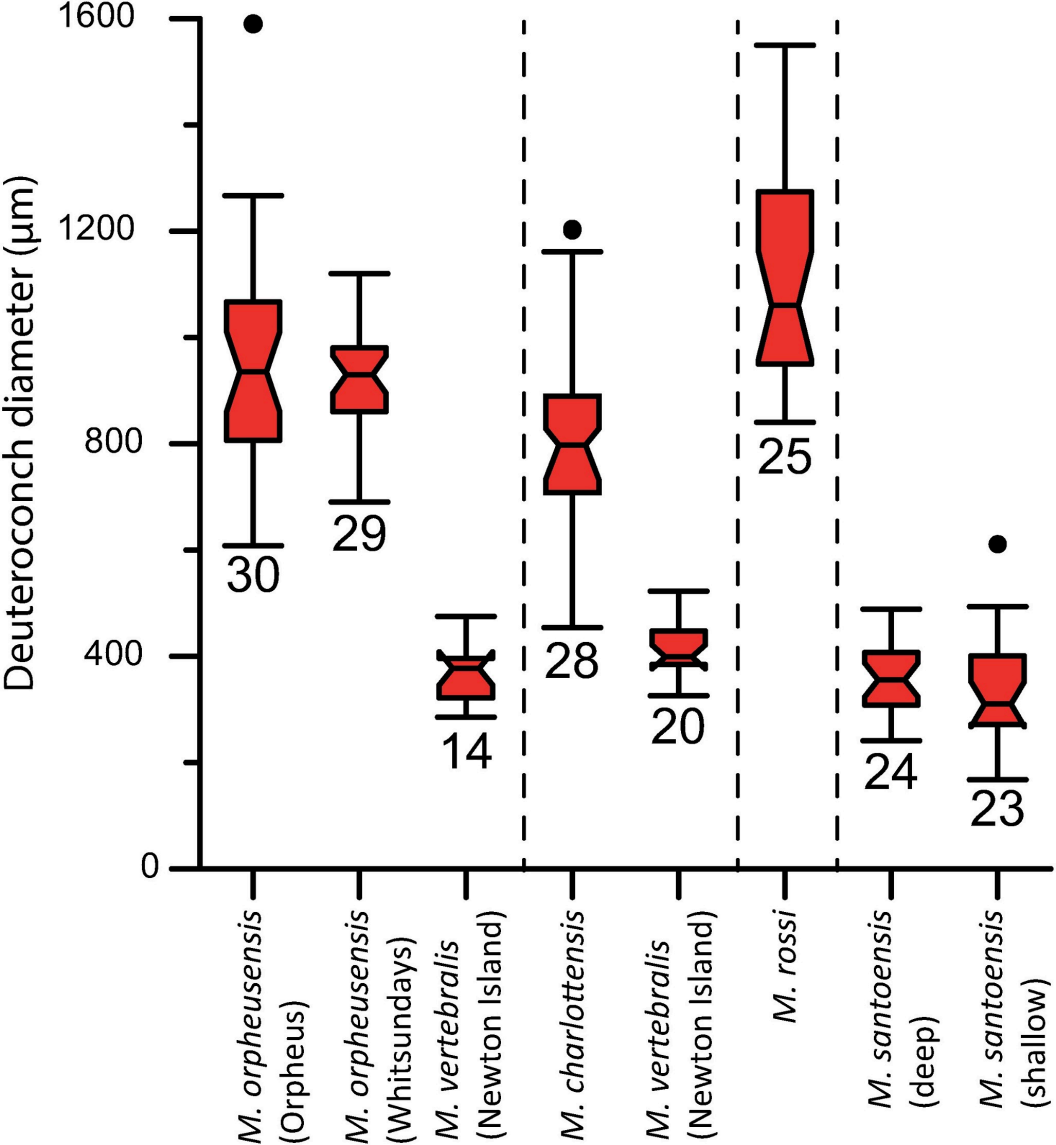
Diameter of the deuteroconch of the A-forms of *M*. *vertebralis*, *M*. *rossi*, *M*. *orpheusensis* nov. spec., *M*. *charlottensis* nov. spec., and deep living (30–40 m water depth) and shallow living (5–15 m water depth) *M*. *santoensis* nov. spec. Boxes represent 1 standard deviation, error bars 2standard deviations, notch is at the median value, and dots represent outliers. Number of specimens is indicated above the error bars.

Diameter of the protoconch 0.1–0.18 mm, deuteroconch: 0.30–0.45 mm (Fig 7), with no differences between the populations from Orpheus Island and Newton Island. In both populations, in 2/3 of the specimens the flexostyle constituted 80–90% (ranging 70–95%) of the outside of the embryonic apparatus. The deuteroconch is connected to the first whorl by a large number of straight radial stolons on regular intervals around the perimeter (Fig 2A).

#### Internal skeleton of the initial chambers

**Fig 8:**
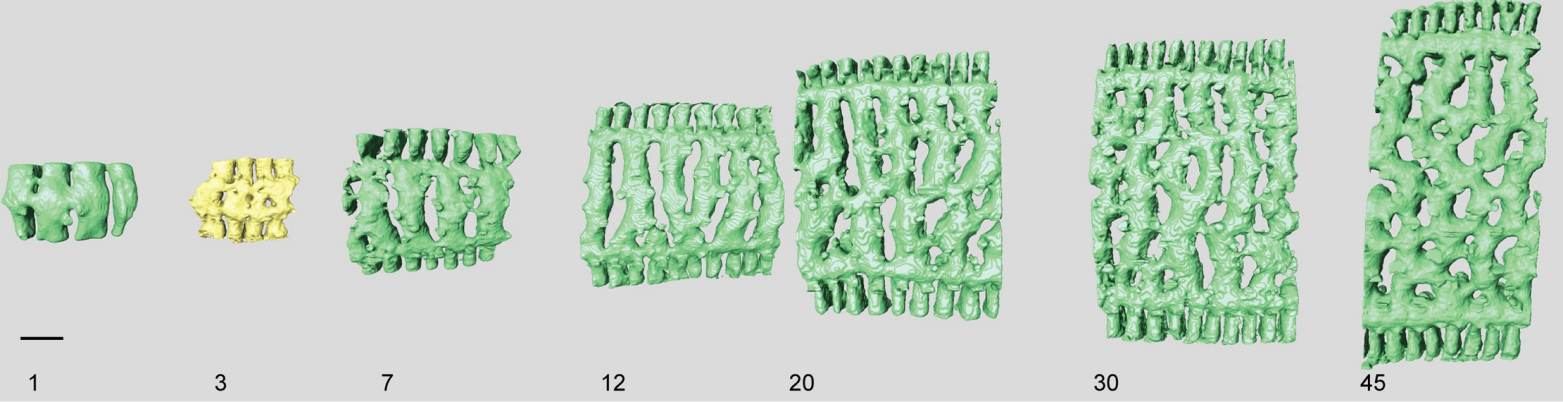
Micro-computed tomography of *Marginopora vertebralis* (RGM.1352105) with the shell removed, showing the interseptal space in chambers 1, 3, 7, 12, 20, 30, and 45. Note the increase in complexity and the frequent occurrence of annular connections in chambers 20, 30, and 45. Scale bar approximately 100 μm.

**Fig 9:**
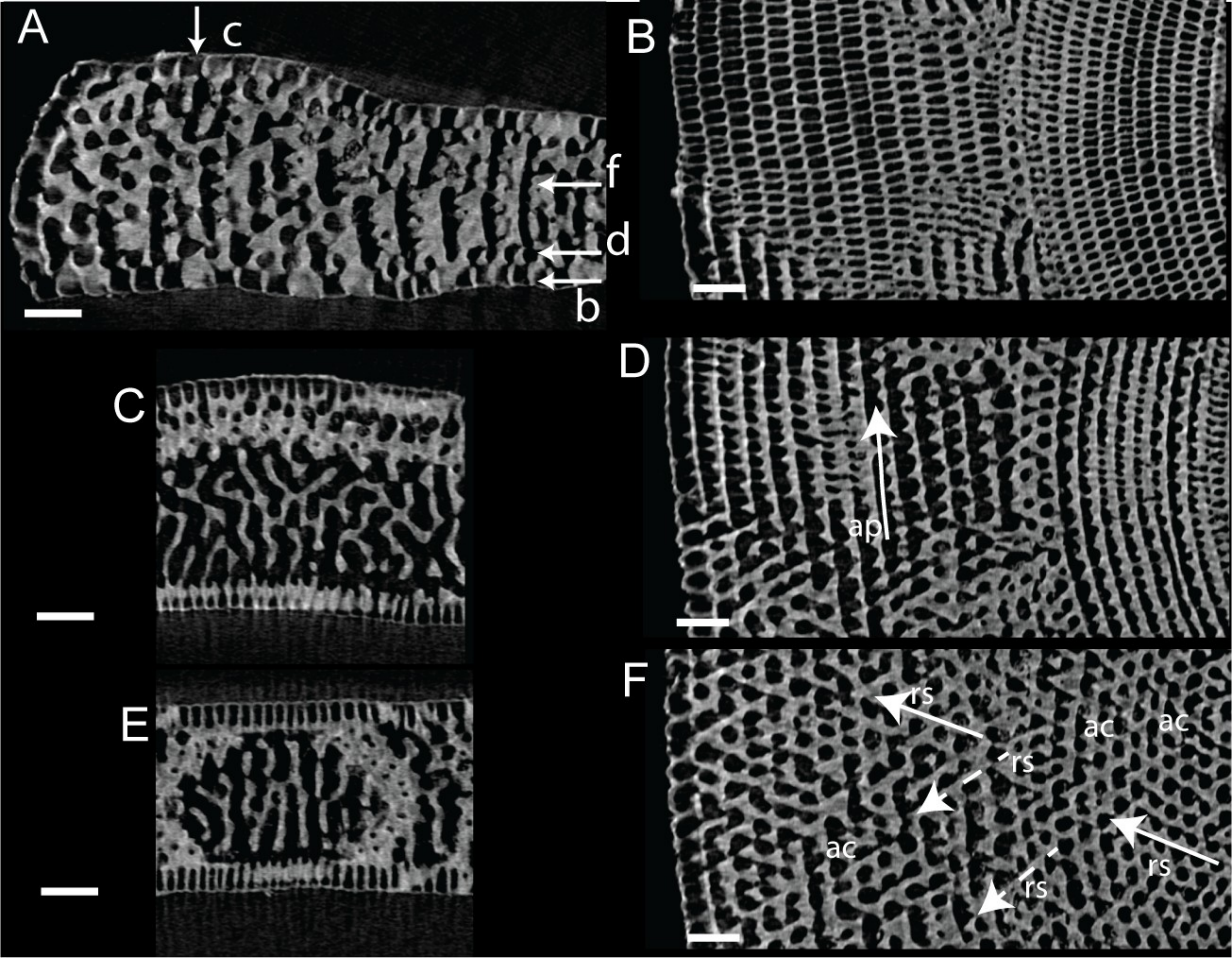
Virtual thin sections of *Marginopora vertebralis* A-form from Newton Island (specimen RGM.1352105). A) Radial virtual vertical thin sections showing the positions of B-F. B) Virtual horizontal section through the lateral chamberlets. C) Virtual vertical thin section perpendicular to A, in the outer part of the shell, showing the bifurcating and irregular interseptal ridges. D) Virtual horizontal section through the annular passage (ap). E) Virtual vertical thin section perpendicular to A, in the inner part of the shell, showing few bifurcations and more regular interseptal ridges than in C. F) Virtual horizontal section through the marginoporid structure showing abundant annular connections (ac) and the alternating directions of the stolon passage (rs). Scale bars 100 μm.

The first chamber consists of the annular passage with chamberlets on either side (Fig 8). The annular passage is divided by septula reaching halfway. Some of these septula cross the entire thickness of the annular passage, portioning it in sections (Figs 8 and 9). In the second chamber the annular passage splits, and in most specimens median chamberlets develop in the third or fourth chamber (Fig 8).

#### Internal skeleton of the outer chambers

Annular passage high, marginal chambers thicker than in any of the other species discussed in this paper (Figs 6 and 8). The number of stolon planes gradually increases. Chamberlets are highly reticulate, and annular connections are common, often connecting three-five chamberlets (Fig 9). The internal skeleton consists of bifurcating, relatively thin interseptal ridges that become increasingly disjunct, sometimes resulting in very rare, flat interseptal pillars in combination with interseptal ridges (Fig 9).

#### Geographical variation in morphology

No morphological differences were observed between the populations from Newton Island and Orpheus Island (Fig 7).

*Marginopora rossi* Lee et al. (2016) [8]

*Marginopora vertebralis* Quoy and Gaimard var. *rossi* Lee et al. 2016 [8]

### Diagnosis

**Fig 10:**
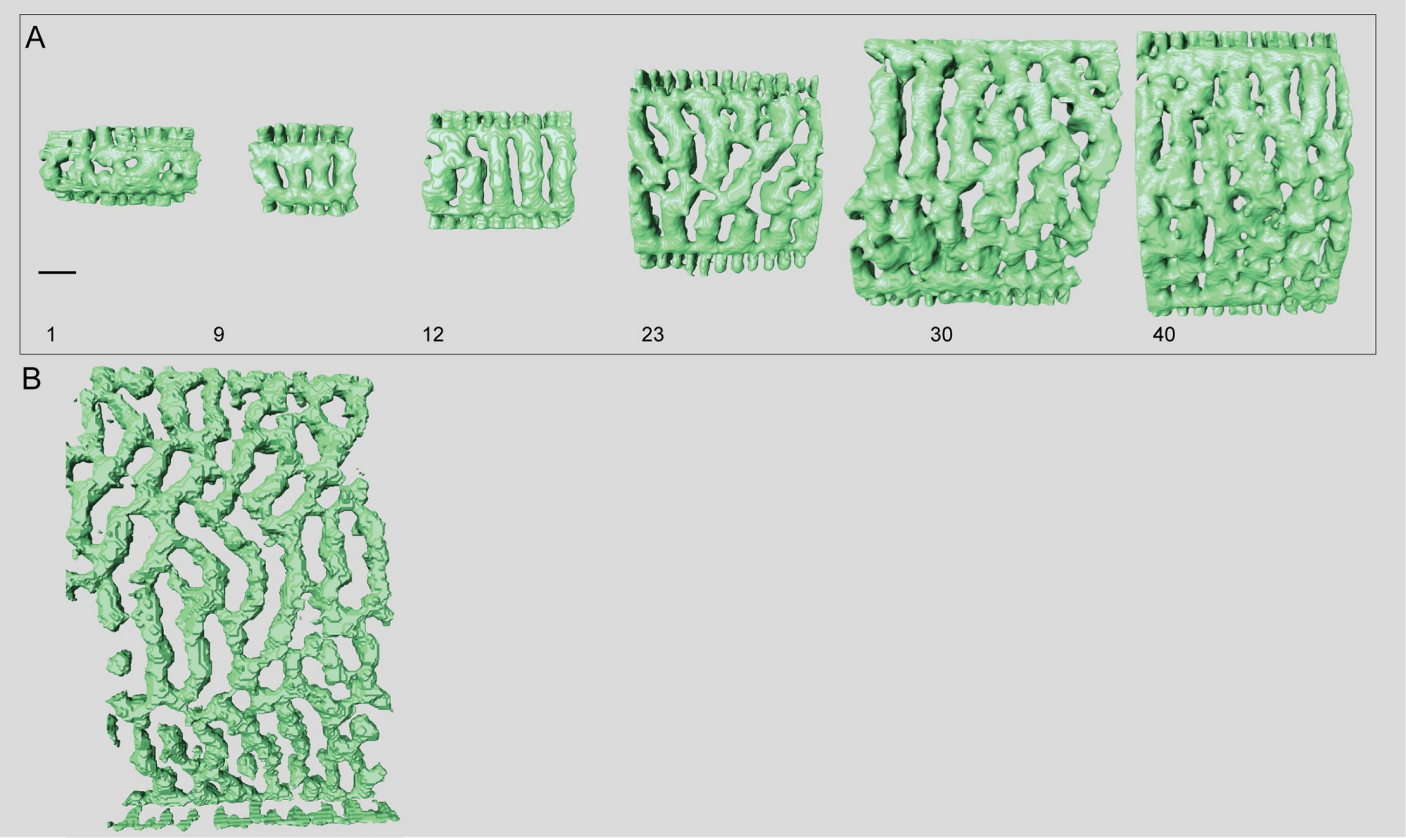
Micro-computed tomography of *Marginopora rossi* with the shell removed, showing the interseptal space. A) chamber 1, 9, 12, 23, 30, and 40 of specimen RGM.1352065; and B) one of the thickest chambers of RGM.1352066. This specimen is missing its central part, so it is not possible to provide an exact chamber number. Note the fully developed chamber structure in chamber 1, as well as the long interseptal ridges even in the thickest chambers. Scale bar is approximately 100 μm).

A large, thick *Marginopora* with Y-shaped stolons (Fig 5) connecting the large deuteroconch with the first annular chamber. Unlike any other known *Marginopora* the first annular chamber has median chamberlets and an annular passage (Fig 10).

### Material

Specimens were collected in July 2008 from the Wistari Channel near Heron Island. 29 specimens were microCT-scanned. RGM.1352063-66.

### Description

#### External morphology

A large, thick shelled *Marginopora*. Thickness increases gradually from the first annular chamber onwards (Fig 3). Growth irregularities occur frequently, and large specimens are often not planar. Microspheric specimens are abundant, and are of similar diameter to the macrospheric specimens. Because the largest specimens do not have the central part of their shells anymore, it is not certain whether there is no size difference between micro- and macrospheric specimens at all. Shells can reach a diameter of up to 25 mm, consisting of ~100 annular chambers, and a thickness of 1.4 mm.

#### Embryonic apparatus

Diameter of the protoconch 0.23–0.35 mm; deuteroconch: 1–1.4 mm (Fig 7). In 26 out of 29 X-rayed specimens the deuteroconch completely envelopes the protoconch and flexostyle (Fig 4), in the other three the proloculus touched the deuteroconch. The deuteroconch is connected to the first whorl by a large number of Y-shaped stolons on regular intervals around the perimeter (Fig 5).

#### Internal skeleton of the initial chambers

**Fig 11:**
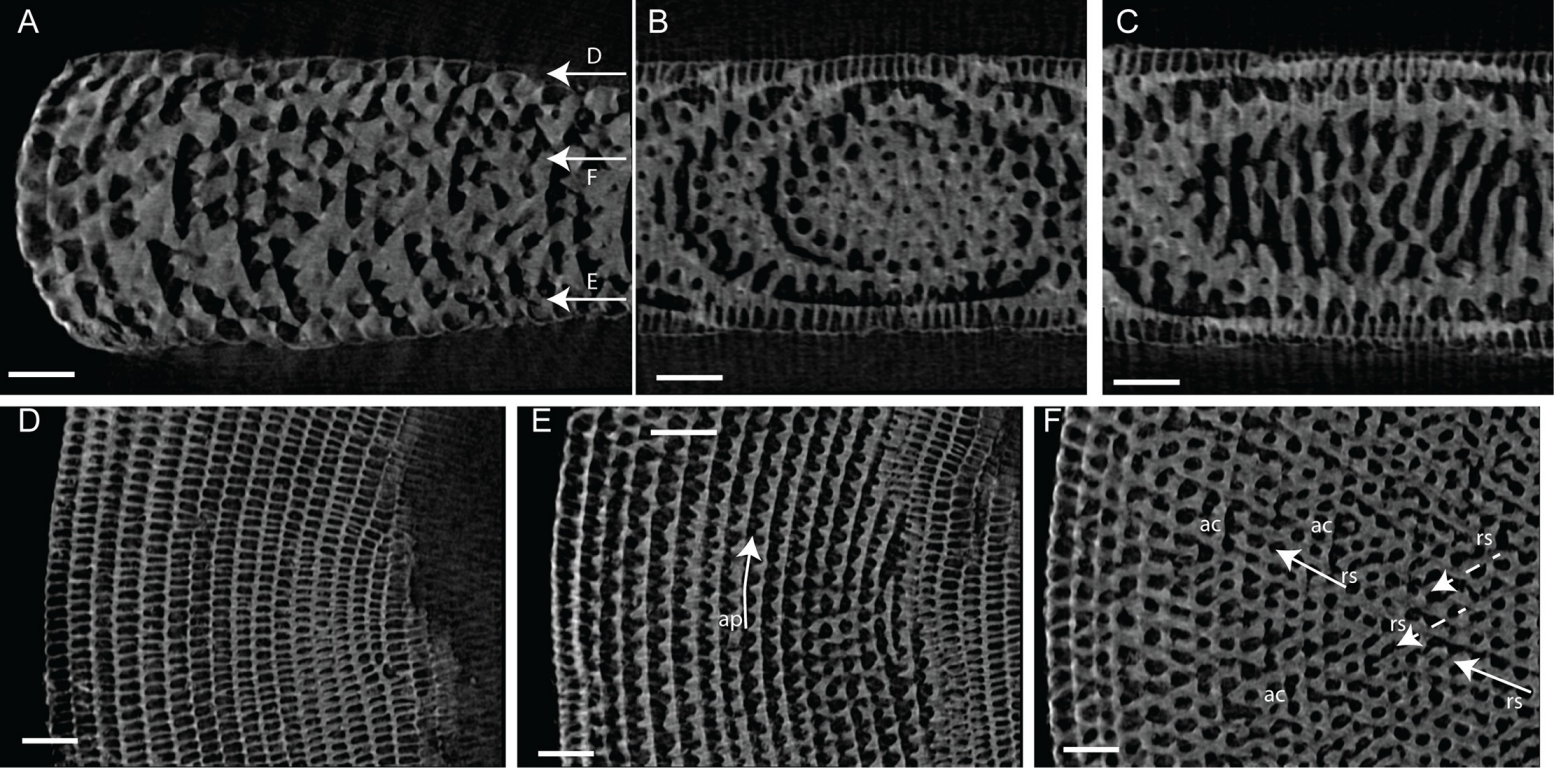
Virtual thin sections of *Marginopora rossi* A-form (RGM.1352063). A: Radial vertical thin section in the outer part of the shell. Arrows indicate the position of D-F. B, C) Virtual vertical thin sections perpendicular to A. B) Chamber wall showing the regularly distributed stolons, which are the apertures when this wall is the outer whorl. C) Interseptal ridges showing irregular placing and infrequent connections. D) Virtual horizontal section through the lateral chamberlets. E) Virtual horizontal section through the annular passage (ap). F) Virtual horizontal section through the marginoporid structure showing rare annular connections (ac) and the alternating directions of the stolon passage (rs). Scale bars 100 μm.

Each of the ends of the Y shaped stolon ends in a median chamberlet, that opens, upwards and downwards, into two annular passages in the first chamber (Fig 10). Marginal chamberlets develop from the annular passage, and are connected by the marginal stolon (the marginal aperture when on the apertural face). Marginal chamberlets are narrow and elongated (Fig 11) and connect to both the previous and active growth increment by the marginal stolon. *M*. *rossi* is the only species in which there are already two rows of median chamberlets in the first whorl (Fig 10). The first chamber is ~190–220 μm high The number of rows of chamberlets gradually increases, and in the outer chambers of the largest specimens there can be as many as 25–30 (Fig 10).

#### Internal skeleton of the outer chambers

Annular passage and marginal chamberlets low (Fig 6). Median chamberlets irregular, reticulate because of frequent annular connections, which only rarely connect more than two adjacent chamberlets (Figs 6, 10 and 11). Chamberlets are constant in diameter. The internal skeleton consists of irregular interseptal ridges that are frequently bifurcating, but no or at most very rare interseptal pillars (Fig 9). In the thickest specimens the frequency of annular connections increases mostly at the marginal parts of the marginoporid structure (Fig 11).

### Remarks

[8] use specimens from the same locality to describe *Marginopora vertebralis* var. *rossi*, which is here erected to species level. The type specimens of [8] are the type specimen for the species.

*Marginopora charlottensis* nov. spec.

### Diagnosis

A medium sized thin and flat *Marginopora* with Y-shaped stolons connecting the large deuteroconch with the first chamber. *Marginopora charlottensis* nov. spec. has the simplest arrangements of the median chamberlets with rare annular connections and mostly parallel interseptal ridges.

### Type specimens

Holotype: Specimen G466207 in the Queensland Museum (Brisbane), field number is JK023_a2; paratypes RGM.1352041-1352055; RGM.1352120, all from the Princess Charlotte Bay area (exact coordinates in the supplementary material). LSID: urn:lsid:zoobank.org:act:54852150-587C-4D7E-AFE5-997E17649F50.

### Derivatio nominis

Named after Princess Charlotte Bay, the area in which the specimens studied herein were found.

### Material

Specimens were collected in October 2007. Nine nearshore-offshore transects were sampled with a van Veen grab between Cooktown to the north of Princess Charlotte Bay. South of Princess Charlotte Bay, *Marginopora charlottensis* nov. spec. is abundant in samples taken between 15–40 m water depth. Microspheres are rare, only three out of >100 specimens checked by X-ray were microspheric. It is not possible to see whether the large specimens without the central chambers are micro- or macrospheric.

### Description

#### External morphology

Intermediate sized, thin, coin-shaped *Marginopora*. (Fig 4). Diameter of the largest specimen is ~20 mm. Specimens are flat and thickness increases slowly, in the first ~13–20 chambers and remains constant around 0.6–0.8 mm (Figs 2 and 3).

#### Embryonic apparatus

Diameter of the protoconch 0.15–0.23; deuteroconch: 0.6–0.8 (0.45–1) mm (Fig 7). In 10 out of 29 X-rayed specimens the deuteroconch partly envelopes the protoconch and flexostyle (Fig 4), in which case the flexostyle touches the deuteroconch wall (Fig 4). The deuteroconch is connected to the first whorl by a large number of Y-shaped stolons on regular intervals around the perimeter.

#### Internal skeleton of the initial chambers

**Fig 12:**
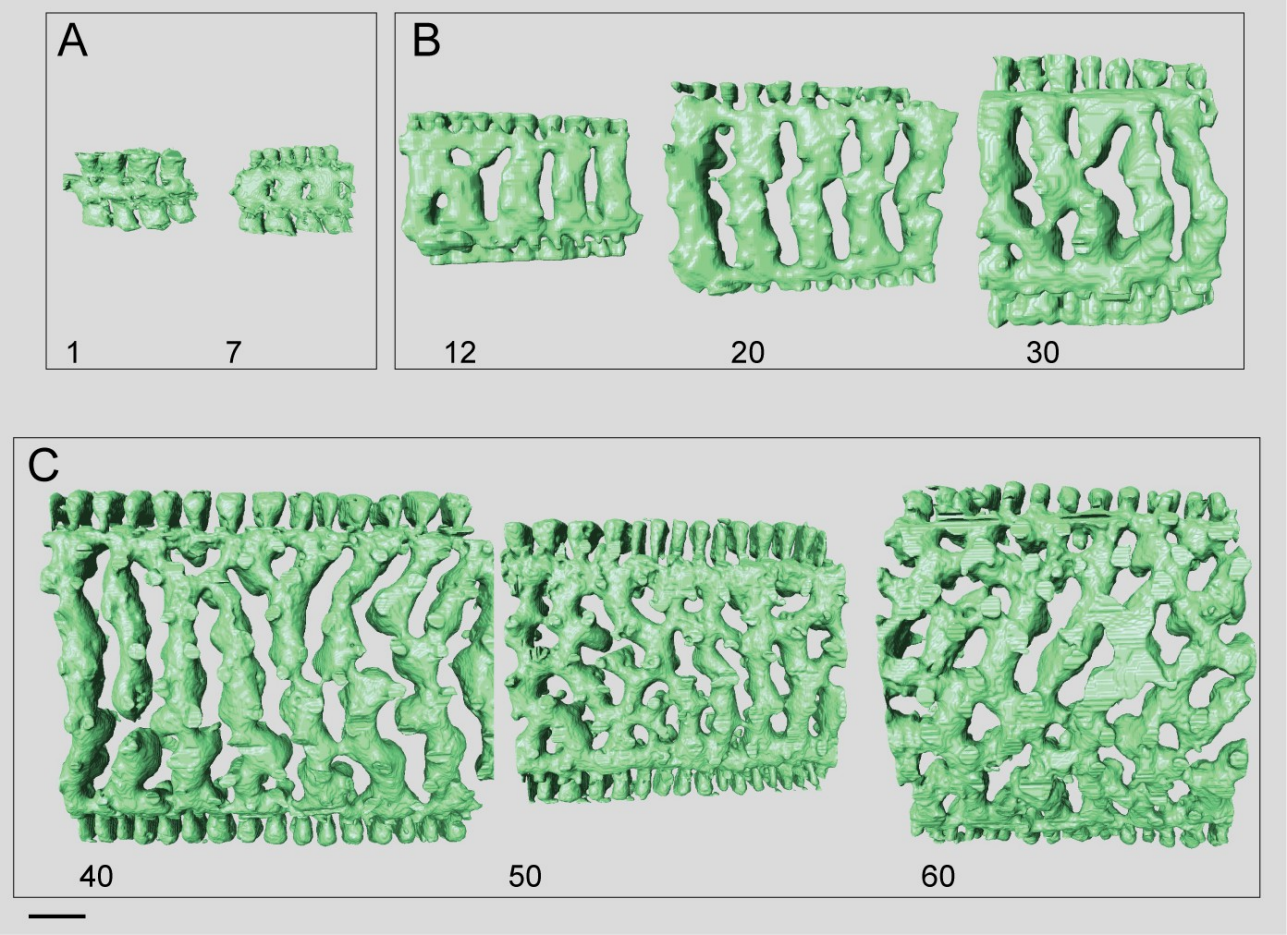
Micro-computed tomography of *Marginopora charlottensis* nov. spec with the shell removed, showing the interseptal space. A) the first and seventh chamber of specimen RGM.1352120; B) the 12^th^, 20^th^, and 30^th^ chamber of specimen RGM.1352054; and C) the 40^th^, 50^th^, and 60^th^ chamber of RGM.132055. Note the very simple structure of the first chamber, the fairly regular and straight chamberlets, and the increasing disjunctions resulting in (flattened) interseptal pillars in the most marginal chambers. Scale bar approximately 100 μm.

The first chamber consists only of the annular passage and with chamberlets on either side (Fig 12). The first chamber is ~100–130 um high In chambers 2–5 the median chamberlets gradually develop, initially by splitting of the annular passage and in most specimens in chamber 7–8 the characteristic internal morphology of the chamber is present (see below).

#### Internal skeleton of the outer chambers

**Fig 13:**
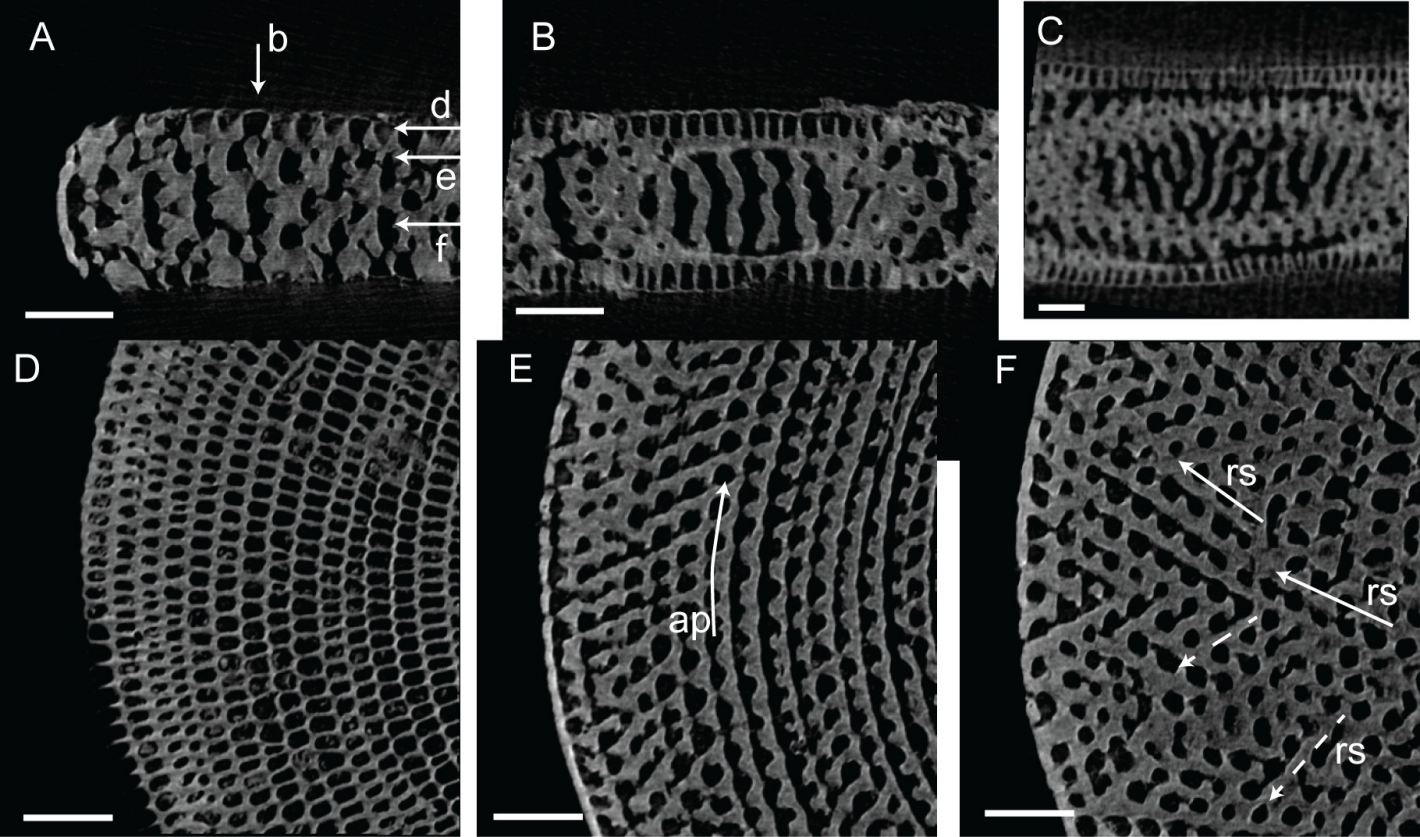
Virtual thin sections of *Marginopora charlottensis* A-form (RGM.1325042). A) Radial vertical thin section in the outer part of the shell. Arrows indicate the position of C-E. B) Virtual vertical thin sections perpendicular to A showing the relatively thick, parallel with no connections nor interruptions. C) Virtual horizontal section through the lateral chamberlets. D) Virtual horizontal section through the annular passage (ap). E) Virtual horizontal section through the marginoporid structure showing rare annular connections (ac) and the alternating directions of the stolon passage (rs). Scale bars 100 μm.

Annular passage and median chamberlets low (Figs 6 and 13). In *M*. *charlottensis* nov. spec. the number of stolon planes and the thickness of the test increases slowly over the first 2 mm radius, when there are usually ~5 stolon panes, which then rapidly increases to 10–12. Chamberlets are slightly oblique to the annular passage, very regular and annular connections are rare, and connect only two adjacent chamberlets. Chamberlets are constant in diameter. The internal skeleton consists of parallel interseptal ridges that rarely bifurcate (Fig 13). In the outermost whorls disjunctions in the ridges become more frequent, resulting in the presence of flattened pillar like structures when the disjunctions are frequent (never round as in *M*. *orpheusensis* nov. spec).

*Marginopora orpheusensis* nov. spec.

### Diagnosis

**Fig 14:**
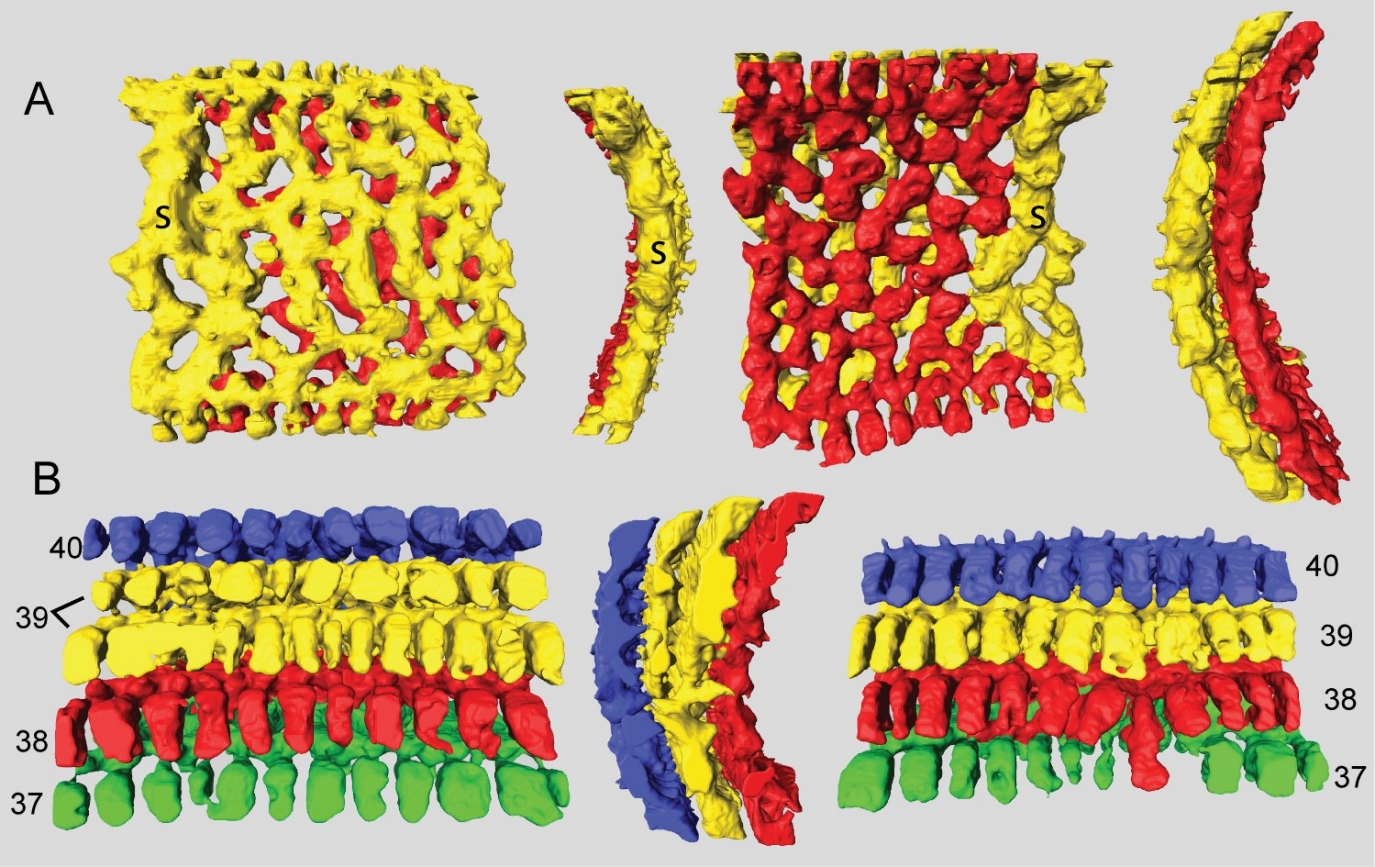
Micro-computed tomography of *Marginopora orpheusensis* nov. spec with the shell removed, showing the interseptal space. A) the 40^th^ chamber of specimen RGM.1352060 showing that a single stack of chamberlets (s) splits into two stacks of chamberlets at least for part of the chamber length, chamberlet height is 0.6 mm) and B) the 37^th^ (green), 38^th^ (red), 39^th^ (yellow) and 40^th^ (blue) chamber of specimen G466208 showing the horizontal doubling of the interseptal space of chamber 39. Note the single double length marginal chamber in the 38^th^ chamber, accompanied by a partial fusion of the annular passages in chamber 37 and 38, suggesting that these chambers combined might be a single growth increment. Chamberlet height is 0.6 mm.

A large sized thick and flat *Marginopora* with Y-shaped stolons connecting the large deuteroconch with the first chamber. In *M*. *orpheusensis* nov spec the derived interseptal morphology is dominated by interruptions of the (mostly) straight interseptal ridges. Uniquely, in later chambers develop first partial, and later complete doubling of the chambers occurs (Fig 14).

### Type specimens

Holotype G466208 (Queensland Museum, Brisbane, Australia), fieldnumber JJ_A3; paratypes: RGM.1352058-61, (fieldnumbers JJ_A1, JJ_A2; Pioneer A1-28). LSID: urn:lsid:zoobank.org:act:EA007DD2-9E7E-47AC-ADBD-608E1BCE6D2C.

### Derivatio nominis

Named after Orpheus Island, the area in which the specimens studied herein were collected.

### Material

Specimens were collected in July 2007. *Marginopora orpheusensis* nov. spec. was found on sand below the reef base, at 12–18 m depth. It was observed at several sites around Orpheus Island at the reef base, but only specimens from Pioneer Bay and Little Pioneer Bay have been included in this study.

### Description

#### External morphology

Diameter up to 28 mm, specimens >15 mm have lost their embryonic apparatus and initial chambers. Coin shaped, flat, gradually increasing in thickness. The largest specimens have up to 80 chambers and attain a thickness of 1 mm. Microspheres are extremely rare, but do not differ from macrospheres other than the morphology of the initial chambers. It is not possible to see whether the large specimens without the central chambers are micro- or macrospheric.

*Embryonic apparatus*. diameter of protoconch 0.23–0.31 mm; deuteroconch: 0.8–1 (0.6–1) mm (Fig 7). In 27 of 29 X-rayed specimens the deuteroconch completely encircled the protoconch and flexostyle (Fig 4). The deuteroconch is connected to the first whorl by a large number of Y-shaped stolons on regular intervals around the perimeter.

#### Internal skeleton of the initial chambers

**Fig 15:**
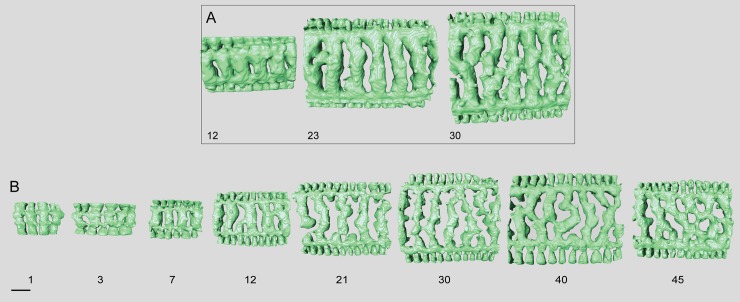
Micro-computed tomography of *Marginopora orpheusensis* nov. spec with the shell removed, showing the interseptal space. A) the 12^th^, 23th, and 30^th^ chambers of specimen RGM.1352058, B) the 1^st^, 3^rd^, 7^th^, 12^th^, 21th, 30^th^ 40^th^, and 45^th^ chambers of G466208. Note the low number of annular connections, and the increase in the number of disjunctions in the interseptal ridges, resulting in a dominance of interseptal pillars in the outermost chambers. Scale bar is approximately 100 μm.

The first chamber consists of a split annular passage with chamberlets to either side, but no median chamberlets nor marginal stolons (Fig 15). The first chamber is ~140–160 um high (Fig 15). From the third or fourth chamber onwards each chamber is differentiated into marginal chamberlets and an annular passage on either side of the median chamberlets (Fig 15). From the fourth whorl onwards the number of rows of median chamberlets increases.

#### Internal skeleton of the outer chambers

**Fig 16:**
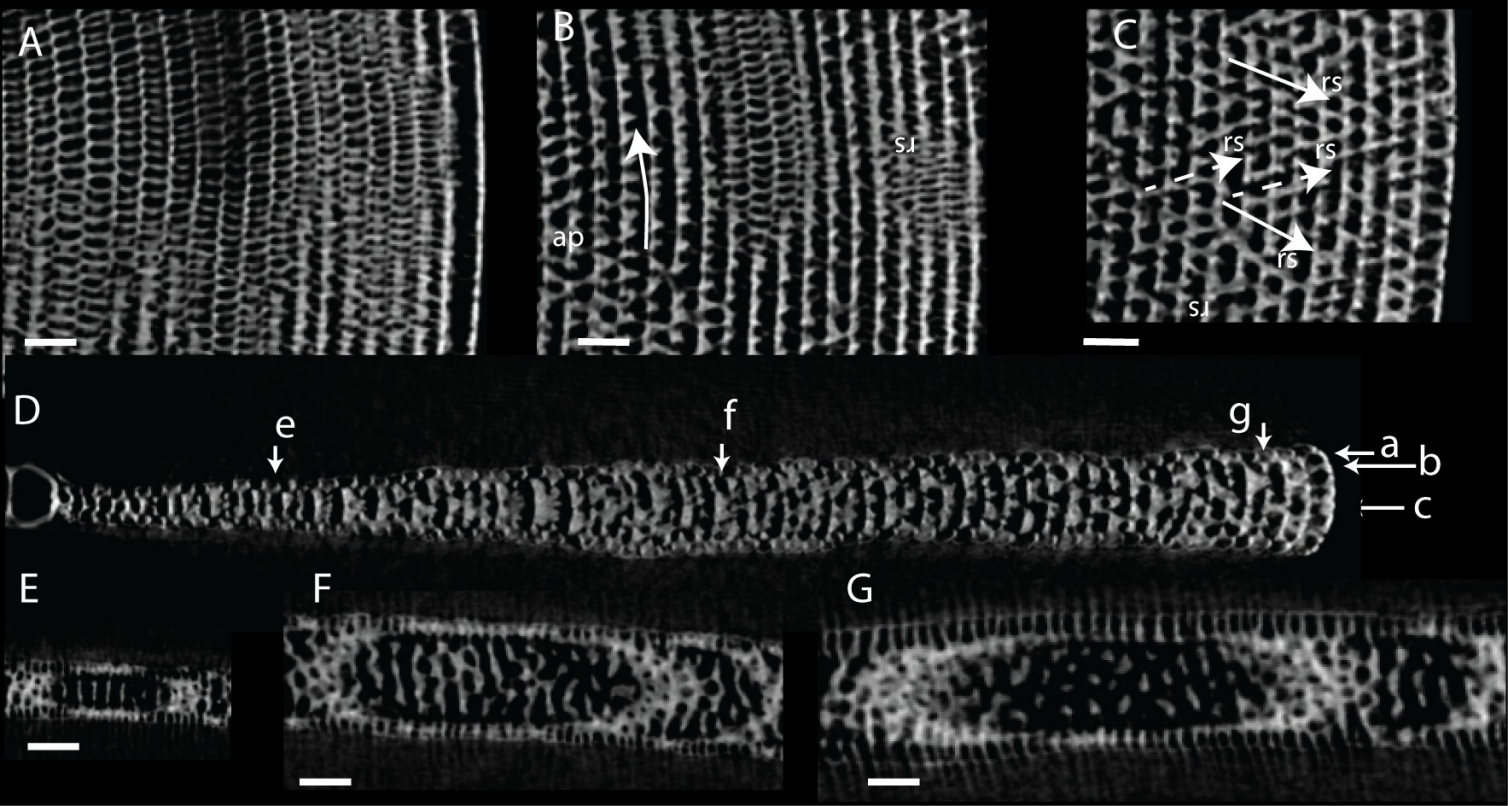
Virtual thin sections of *Marginopora orpheusensis* nov. spec. A-form (RGM.1352061). A) Virtual horizontal section through the lateral chamberlets. B) Virtual horizontal section through the annular passage (ap). C) Virtual horizontal section through the marginoporid structure showing rare annular connections (ac) and the alternating directions of the stolon passage (rs). D) Radial vertical thin section. Arrows indicate the position of A-C and E-G. E) Virtual vertical thin section perpendicular to D showing the straight interseptal ridges in the initial chambers. F) Virtual vertical thin section perpendicular to D showing the increased complexity, especially by increasing the number of disjunctions in the interseptal ridges in the middle chambers. G) Virtual vertical thin section perpendicular to D showing the interseptal pillars in the outer chambers. A-C, E-G Scale bar 100 μm; D: 500 μm.

Annular passage low, and marginal chamberlets narrow. The chamberlets are straight, separated by interseptal ridges (Fig 16). Complexity in the structure arises from an increasing number of discontinuities, rather than bifurcations (Fig 15). In most specimens the final 10–20 chambers are thinner than the previous chambers. In the transitional chambers, an additional wall parallel to the chamber wall is formed (Fig 14). In other specimens similar doublings are observed along horizontal axes, in which case a single annular passage connects to two stacks of median chamberlets (Fig 14). In this area the interseptal ridges gradually are replaced by interseptal pillars, as the result of the increasing number of discontinuities (Figs 15 and 16).

*Marginopora santoensis* nov. spec.

### Diagnosis

A flat *Marginopora* with I-shaped stolons connecting the deuteroconch with the first chamber (Fig 5). The combination of a, relatively, small deuteroconch with a very simple arrangement of the median chamberlets with rare annular connections is unique.

### Type specimens

Holotype RGM.1352067; paratypes: macrospheric specimens: RGM.1352121-2; RGM.1352068-100; microspheric specimens: RGM.1352117-119. LSID: urn:lsid:zoobank.org:act:93ED1A37-E977-40A9-A5ED-F8609DDDDFC4

### Derivatio nominis

Named after Esperitu Santo (Vanuatu), the island where the specimens studied herein were collected.

### Material

Numerous specimens collected from 0.5–40 m depth around the SE coast of Esperitu Santo (Vanuatu), collected in October 2006. Shallow-living populations occurred in high densities on rubble or macro-algal substrates. Deep living specimens could also be observed on sand.

### Description

#### External morphology

A- forms vary from coin-shaped, flat, gradually increasing in thickness towards the margin (Figs 2 and 3). In large specimens the maximum thickness of the test is in the second third of the test radius, and tapers towards margin (Fig 3). Maximum diameter of the macrosphere is ~5 mm with a thickness of 0.25 mm in thin specimens and 0.5 mm in thick specimens. Microspheres are flat to mildly undulated, and can have a diameter of ~12 mm. Also within the microspheres there is considerable variation in the thickness of the test. Maximum thickness is about ~0.8 mm.

#### Embryonic apparatus

Diameter of the protoconch 0.11–0.15 (0.1–0.2) mm; deuteroconch: 0.35–0.4 (0.3–0.5) mm (Fig 7), with an outlier of 0.65 mm. The deuteroconch is connected to the first whorl by a large number of straight radial stolons on regular intervals around the perimeter (Fig 5C). The deuteroconch envelopes 70–90% of the flexostyle and protoconch (Fig 4).

#### Internal skeleton of the initial chambers

**Fig 17:**
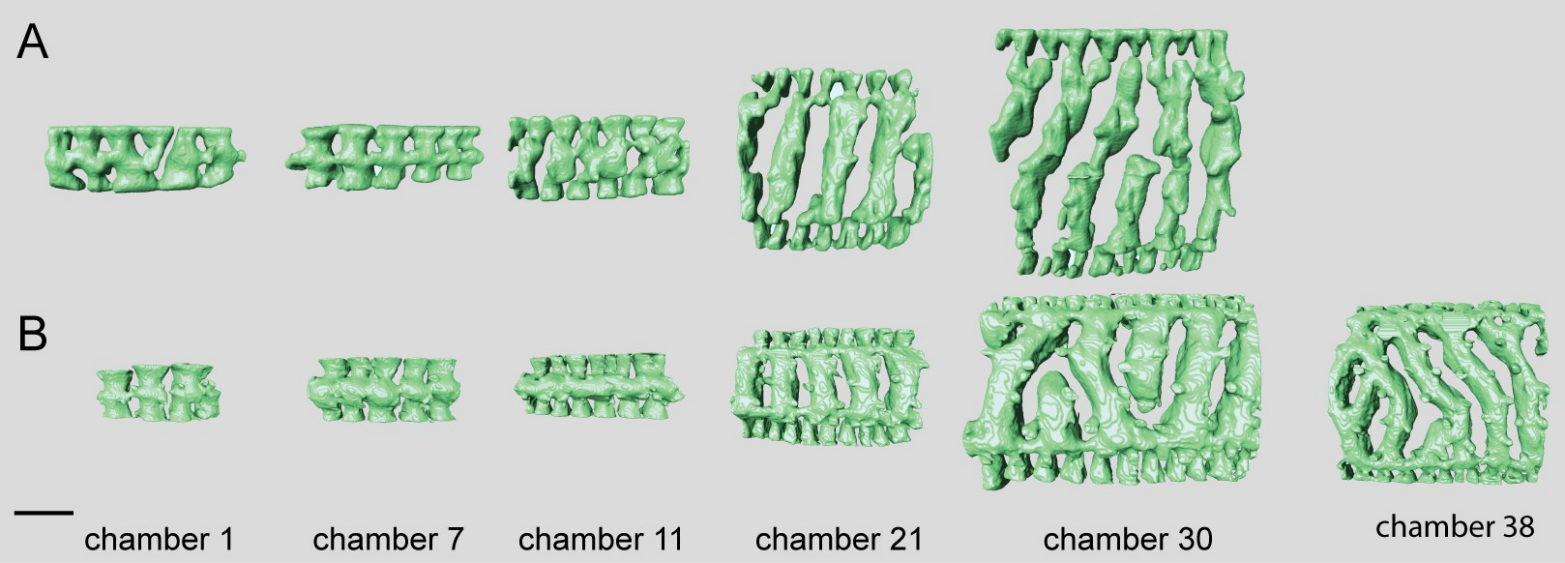
Micro-computed tomography of *Marginopora santoensis* nov. spec with the shell removed, showing the interseptal space. A) the first, seventh, 11^th^, 21th, and 30^th^ chamber of specimen RGM.1352068; B) the 7^th^, 11^th^, 21th, 30^th^ and 38^th^ chamber of specimens RGM.1352070. Note the increase in complexity within specimens, and the similarity in the order of changes between the specimens, but in the thicker specimen RGM.1352068) these changes occur in earlier chambers. Scale bar is approximately 100 μm.

**Fig 18:**
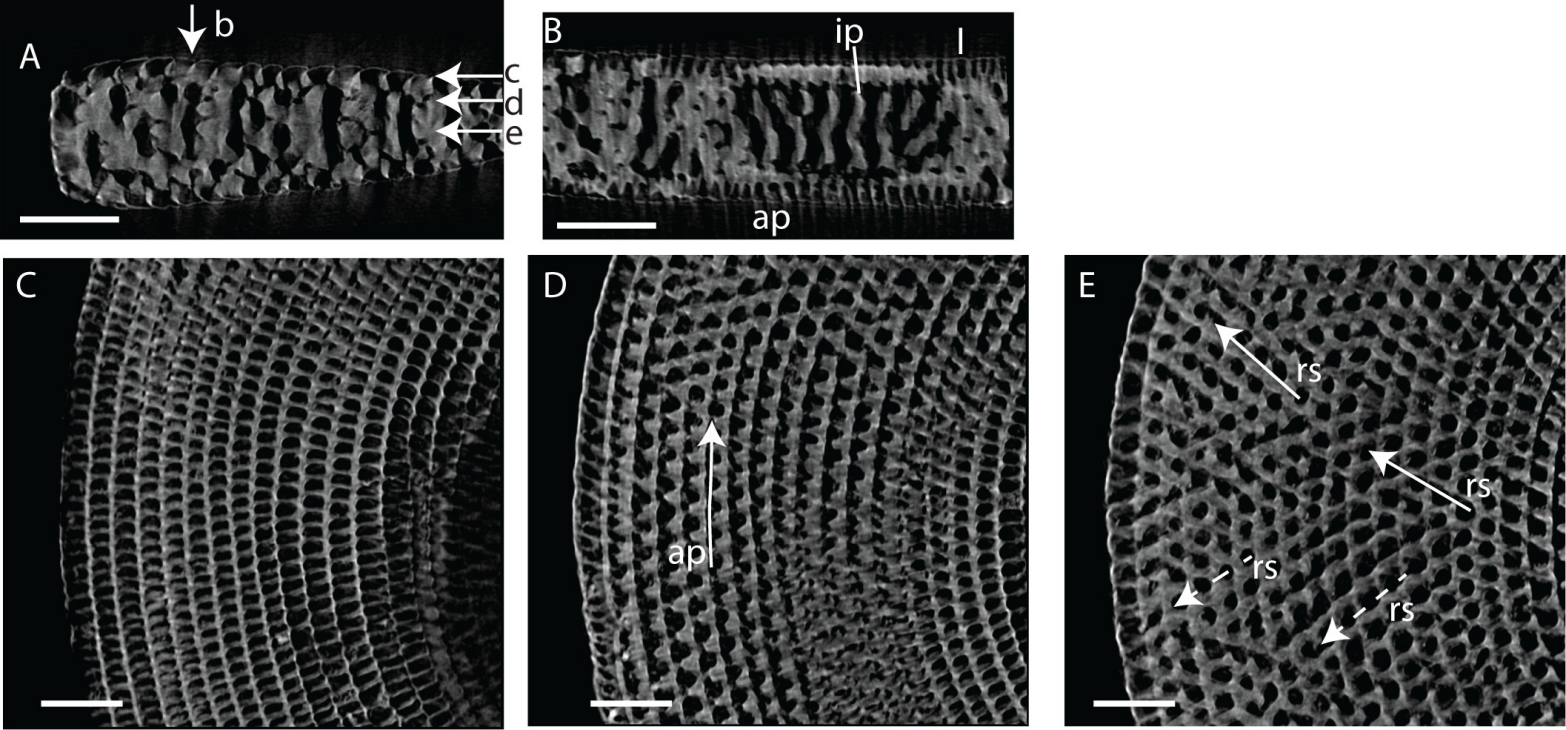
Virtual thin sections of *Marginopora santoensis* nov. spec. A-form (RGM.1352067). A) Radial vertical thin section in the outer part of the shell. Arrows indicate the position of B-E. B) Virtual vertical thin section perpendicular to A showing the irregular interseptal ridges with few connections and rare disjunctions. C) Virtual horizontal section through the lateral chamberlets. D) Virtual horizontal section through the annular passage (ap). E) Virtual horizontal section through the marginoporid structure showing rare annular connections (ac) and the alternating directions of the stolon passage (rs).

In all specimens the first chamber consists of the annular passage with chamberlets on either side (Fig 17). The annular passage is divided by septula reaching halfway the chamberlet (Figs 17 and 18). Some of the septula cross the entire thickness of the annular passage (Fig 17). In thin specimens the annular passage splits in the 10^th^-11th chamber, in thicker specimens this happens in the 7^th^-8th chamber. In thin specimens the median chamberlets develop in the 12^th^ -16^th^ chamber. In thick specimens this occurs in the 8-10th chamber (Fig 17).

#### Internal skeleton of the outer chambers

Annular passage as high as the height of the marginal chamberlets (Figs 17 and 18). In thin specimens there are ~5 rows of median chamberlets, in thick specimens 9 at a radius of 2–3 mm after which the thickness, and number of stolon planes decreases. In the larger microspheres there are also at most 10–11 rows of median chamberlets. There are very few annular connections. The internal skeleton consists of parallel interseptal ridges. In thicker specimens the interseptal ridges are more irregular and sometimes bifurcate. Annular connections are rare, not more than two adjacent chamberlets.

### Differences between species

*Marginopora rossi*, *M*. *charlottensis* nov. spec. and *M*. *orpheusensis* nov. spec. all are large and flat and live on sandy substrates on the reef base and inter reef (Fig 2). They share a morphological feature not observed in any other *Marginopora* species, which is the large deuteroconch with Y-shaped stolons. Additionally, each of the species has a unique set of characters. Several of the characters discussed here have also been related to environmental variation, such as the thickness of the shell. Others, such as diameter of the deuteroconch, have frequently been used to characterize species, especially in the fossil record. Although complexity of the chamberlets in all species is related to thickness of the shell, and thus with ontogeny, each of the three populations has unique features rarely seen in the others. For example, even in the thickest chambers of *M*. *rossi* the interseptal space is dominated by ridges and bifurcations (Fig 10), in *M*. *charlottensis* nov. spec. and especially *M*. *orpheusensis* nov. spec. the interseptal space of the final chambers is dominated by interseptal pillars (round in *M*. *orpheusensis* nov. spec., flattened in *M*. *charlottensis* nov. spec.). In combination with the equally consistent differences in the morphology of the first chamber, each of the species has a unique set of characters which is independent of variations in shell thickness or deuteroconch diameter. Based on these observations I conclude that this are three probably closely related species, different from *M*. *vertebralis* living on the reef flat.

## Discussion

Distinct morphological differences were used to recognize five species of *Marginopora*, interpreted to belong to two previously described species and three new species. In two occasions, two species were observed in a single reef system, but in non-overlapping habitats, which demonstrates that these species can co-exist in close proximity while remaining morphologically different. The shallow-living reef crest species, *M*. *vertebralis*, is morphologically homogeneous throughout the Great Barrier Reef, with populations from the North, Central, and South Great Barrier Reef having the same characteristics (Fig 7). This suggests that in the Great Barrier Reef there is a single widely distributed species living on the reef flat and reef crest. Morphological differences between the Wistari channel (Heron) and reef flat/crest population at Lizard Island [8] include, among others, the tendency to develop more annular connections between median chamberlets in the marginoporid structure in the shallow living populations. For the reef base Heron island population, *M*. *rossi*, [8] reported a few specimens with the embryonic apparatus intact, in which the same configuration as described in this paper with the deuteroconch enveloping the protoconch and flexostyle was observed. The larger size of the protoconch and embryonic apparatus is not mentioned, however. Other populations found at the reef base of in the Central Great Barrier Reef (Orpheus Island) and North Great Barrier Reef (Lizard Island) differ from *M*. *vertebralis* by their large embryon and vorhof (Fig 7), the vorhof completely or almost completely envelopes the proloculus and flexostyle (Fig 3A, 3B, 3D and 3F), and the Y-shaped stolons between the deuteroloculus and the first annular chamber (Fig 2). Based on these characters, the three reef base populations studied here are more similar to each other, than to the reef crest *M*. *vertebralis*. However, each of the three populations have unique morphological characters that have been interpreted as being important at the species level.

Large benthic foraminifera usually are associated with a large degree of morphological variation based on the trade-off between light intensity and hydrodynamic energy, both along depth gradients [19], in experimental conditions [20], and in onshore-offshore gradients [21]. With the exception of the reef crest *M*. *vertebralis*, the Great Barrier Reef populations all consist of single populations or when multiple samples are available from single environments, and thus are not suitable to estimate the extent of phenotypical variation within the populations. However, *M*. *santoensis* nov. spec. has been found in a wide range of environments and depths, and this species is used here to quantify the characters in *Marginopora* which are especially affected by environmental conditions. These specimens have been collected in shallow habitats with high hydrodynamic energy and in deep habitats with less hydrodynamic energy. Analysis of the internal structure of *M*. *santoensis* nov. spec., shows that the morphology of the embryonic apparatus is similar to *M*. *vertebralis*, whereas the median chamberlets at the thickest part of the test is most comparable to *M*. *rossi*. Similar to other larger benthic foraminifera, specimens living in high hydrodynamic energy are thicker [19–21]. The skeletal morphology of the later chambers is comparable between shallow and deep living specimens. A similar number of median chamberlets is found at comparable thickness of the test, and as a consequence the addition of new rows of median chamberlets occurs in earlier chambers in thick specimens. Rare bifurcations occur more frequent in chambers with more median chamberlets. In specimens with the same number of median chamberlets, however, the interseptal ridges are of the same shape. Thus, environmental conditions affect the morphology of *Marginopora* shells, but the variation is smaller than the morphological differences between the populations discussed herein.

A complicating factor in comparing morphological differences between populations is that, next to ecophenotypical variation also ontogenetic variation is a factor to take into account [6]. From early to later chambers the structure tends to become more complex. In most taxa the initial chambers only consist of a single annular passage with chamberlets on either side. These chamberlets resemble marginal chamberlets, but are structurally different because they are not connected the annular passage of the next chamber, as is the case in marginal chamberlets. In later chambers the annular passage splits into two, but without the marginoporid structure being present yet. Only once the median chamberlets develop, the typical differentiation within each chamberlet is present. In the initial chamber with this structure, usually the chamberlets are straight, and only in later stages the more complex structures develop. The rate at which these ontogenetic changes occur, differs between the species. *Marginopora rossi* is the only species that already has median chamberlets in the initial chamber. At the other extreme is *M*. *santoensis* nov. spec., in which there are at least 12–15 chambers with only the annular passage and chamberlets on eiter side. From chamber 15–20 the median chamberlets are present as well. Whereas in *M*. *rossi*, and *M*. *santoensis* nov. spec. structural complexity increases by an increasing number of bifurcations of the chamberlets, in *M*. *charlottensis* nov. spec. and *M*. *orpheusensis* nov. spec., especially in the last chambers, the number of disjunctions increases, resulting in an interseptal skeleton of pillars (round in *M*. *orpheusensis* nov. spec., flattened in *M*. *charlottensis* nov. spec.). In conclusion, there is also a distinct difference in the ontogenetic pattern between each of the species described herein.

### Morphology conclusion

In contrast, in Vanuatu occurs a single morphospecies, in which shallow and deep living populations are morphologically identical (Fig 7).

### CSI *Marginopora*: Linking morphology to available genetic data

Soritidae, and especially the genus *Marginopora* have been the focus of numerous molecular phylogenetic studies (e.g. [3, 8, 12, 13]).

#### Evidence in favor of a single widespread reef crest species

The oldest molecular study on *Marginopora* is by Benzie and Pandolfi [11] who found no significant allozyme differentiation in *M*. *vertebralis* populations over the entire Great Barrier Reef. Differences between populations were structure by distance between populations and depth of the water separating these populations [11]. All specimens in [11] were collected at less than 4 m depth (Table 1 in [11]), consistent with the morphological evidence of a single species occurring in reef crest and reef flat environments across the entire Great Barrier Reef. These findings are supported by [13] who also collected specimens in the shallowest 2–4 m depth at reefs from the northern Great Barrier Reef to as far south as Lord Howe Island. They found that all specimens, apart from a single specimen from Fiji, belong to a single phylotype (Mar II as defined in [12]). My new sequences from *M*. *vertebralis* at Orpheus Island are identical to these sequences.

The latter are the only specimens in this phylotype for which both morphological and molecular data are available. From several of the populations studied by [13] specimens from the same sites and sometimes samples, provided by Sven Uthicke, show the same morphological characters as the largest population of *M*. *vertebralis* I studied from Newton Island (Fig 7).

In contrast, data on the deeper living taxa, *M*. *rossi*, *M*. *orpheusensis* nov. spec., and *M*. *charlottensis* nov. spec., are more scattered. The Mar III phylotype of [12] was collected from the reef base at Lizard Island. In absence of specimens for which both genetic and morphological data ae available, it can only be inferred that *Marginopora charlottensis* nov. spec. compares to specimens from this phylotype. This species was abundant in inter reef sediments from around Lizard Island, and thus observed in close proximity of the locality of the specimens sequenced by [12].

*Marginopora rossi* was sequenced by [8] but these data are not available. They reported that their sequences from the Heron Island population (the type locality of *M*. *rossi*) also group with phylotype Mar III, however, all sequences from Heron are more similar to each other than to the specimens from Lizard Island ([8], [12]).

From a single area genetic data are available for both the shallow-living and deep-living populations. Momigliano and Uthicke [13] sequenced shallow living *M*. *vertebralis* from Lizard Island and [12] sequenced reef base specimens collected at 30 m deep from the same island. This site is next to the type locality of *M*. *charlottensis* nov. spec., and most likely the same taxonomic unit. The difference between *M*. *charlottensis* nov. spec. and *M*. *vertebralis* from Lizard is genetically distinct (Fig 1).

Thus, both available morphological as well as genetic evidence suggests consistent differences between *M*. *vertebralis* and the inter reef populations. Morphological evidence suggests that these inter reef populations represent at least three species, one in the north Central, one in the South Central and one in the Southern Great Barrier Reef. Although the presence of three species in clade Mar III is not disproven by the available molecular phylogenetic evidence, differences cannot be tested because the sequences of *M*. *rossi* [8] are not available from gen bank.

These findings are in contrast to [8] who concluded that *Marginopora* is a monotypic genus, partly using the same populations as in this study. This is surprising since in previous studies on foraminifera, morphologically defined species often show considerable genetic diversity, which may be indicative of the existence of cryptic species within morphospecies ([22] and references therein). In conclusion, both morphological and genetic data support a shallow and a deep living clade of *Marginopora* on the Great Barrier Reef.

One or more species in phylotype MarIII? The remaining question is whether the MarIII (*M*. *rossi/M*. *orpheusensis* nov. spec. */M*. *charlottensis* nov. spec. complex*)* phylotype consists of a single widespread or multiple narrow ranging species. Structural differences between the three species are consistent, and each of the species has unique morphological characters. This is in contrast with previous studies on foraminifera, where morphologically defined species often show considerable genetic diversity, which may be indicative of the existence of cryptic species ([22] and references therein). In the MarIII phylotype, this appears to be the other way around, distinct morphological differences are only weakly supported by molecular evidence.

There are a number of possible explanations for this pattern: 1) Insufficient genetic data. Each population was sampled and analysed in different studies. As a consequence length of the obtained sequences differ between studies. 2) Genetic differences really are very small between populations. One of the possible mechanisms behind this could be that the MarIII phylotype species complex is very young and did not have enough time to genetically differentiate. A possible mechanism for this is that during the Last Glacial Maximum sea-level minimum the entire Great Barrier Reef was exposed to air, and coastal organisms (including large benthic foraminifera and corals) were restricted to relatively small areas outside their current geographic range. Originally it was assumed that during the deglaciation these were repopulated from Coral Sea plateau reefs [23–25]. For the zooxanthellate coral *Acropora tenuis* and *A*. *millepora* a genetic break was observed at around 19°N, while the limit between the range of *M*. *charlottensis* nov. spec. and *M*. *orpheusensis* nov. spec. is somewhere between 14 and 19°N. This genetic break was interpreted as an result of admixture of alleles by the SE Australia current [26]. Recent observations of reef refugia along the Great Barrier Reef shelf edge, some of which could serve as refugia for inter reef species as well, could indicate a recolonization from the Great Barrier Reef shelf edge, resulting in population bottlenecks and potentially speciation [27]. The deep-living populations have extremely large embryonic apparatuses (larger than the complete test of most foraminifera) and likely disperse over short distances.

## Conclusion

In this study I find additional structural differences between the shallow *M*. *vertebralis* and deep water Great Barrier Reef species (*M*. *rossi; M*. *charlottensis* nov. spec, *M*. *orpheusensis* nov. spec.), that were not recognized before. Eye catching are the bifurcating radial stolons in the deuteroconch wall, the diameters of the protoconch and deuteroconch, and the less abundant annular connections between chamberlets in the same stolon plane. Additional, admittedly circumstantial, evidence that there is a difference at the species level between deep and shallow-living *Marginopora* is that *Marginopora vertebralis* occurs in reef crest and reef flat environments over large parts of the Pacific [28], whereas the deeper living species are restricted to the Coral Sea. If the discussed morphological differences are ecophenotypic variation within a single species, you would expect that both forms could occur in the right environment over its the entire geographic range. However, I am aware of deep living *Marginopora* populations only from the entire Great Barrier Reef, Vanuatu, and New Caledonia.

Given all these considerations, I interpret the populations described here as different species. Available molecular evidence supports this interpretation (Fig 1C). However, molecular evidence from *M*. *orpheusensis* nov. spec. and *M*. *santoensis* nov. spec. should confirm these conclusions. Based on the molecular phylogeny [8, 13] the consequence of interpreting the Newton and Heron populations as distinct at the species level, is that, to maintain monophyletic species, the Guam population should also be seen as distinct at the species level as well [12]. I have not been able to study the morphology of this population. All of the populations studied here showed unique combinations of character states. Size of the deuteroconch, I or Y shaped stolons connecting the deuteroconch with the first chamber, and the presence and shape of interseptal ridges or pillars are the important characters. Although shells tend to become more complex with increasing number of whorls, the character combinations discussed here are persistent from early whorls on. In any given sample only a single morphotype is found. In conclusion, rather than being a monotypic genus, there are probably several species of *Marginopora*, several of which have a rather narrow geographic distribution.

If this interpretation is correct, the genus *Marginopora* in the Coral Sea is represented by a single wide ranging reef crest species, and at least four reef base/inter reef species with a much narrower geographic distribution. This could reflect the presence of reef refugia during past glacials.

These conclusions could be tested by performing a molecular phylogenetic studies including each of the populations in a single tree. This study also highlights the importance of making sequences available in public databases upon publication.

## Supporting information

S1 Table Morphological measurements used in this study. (XLSX)
